# Can we accurately report PTEN status in advanced colorectal cancer?

**DOI:** 10.1186/1471-2407-14-128

**Published:** 2014-02-25

**Authors:** Christopher Hocking, Jennifer E Hardingham, Vy Broadbridge, Joe Wrin, Amanda R Townsend, Niall Tebbutt, John Cooper, Andrew Ruszkiewicz, Chee Lee, Timothy J Price

**Affiliations:** 1 The Queen Elizabeth Hospital, TQEH Woodville Road, Woodville South, SA 5011, Australia; 2 Bazil Hetzel Institute, Woodville Road, Woodville, SA, Australia; 3 University of Adelaide School of Medical Sciences, Adelaide, Australia; 4 University of Adelaide School of Medicine, Adelaide, Australia; 5 Austin Health, Melbourne, Victoria, Australia; 6 Australasian Gastrointestinal Trials Group, Sydney, NSW, Australia; 7 SA Pathology, Adelaide, Australia; 8 Clinical Trials Centre, University of Sydney, Sydney, NSW, Australia

**Keywords:** PTEN, Colorectal, Immunohistochemistry, Copy number, Mutation

## Abstract

**Background:**

Loss of phosphatase and tensin homologue (PTEN) function evaluated by loss of PTEN protein expression on immunohistochemistry (IHC) has been reported as both prognostic in metastatic colorectal cancer and predictive of response to anti-EGFR monoclonal antibodies although results remain uncertain. Difficulties in the methodological assessment of PTEN are likely to be a major contributor to recent conflicting results.

**Methods:**

We assessed loss of PTEN function in 51 colorectal cancer specimens using Taqman® copy number variation (CNV) and IHC. Two blinded pathologists performed independent IHC assessment on each specimen and inter-observer variability of IHC assessment and concordance of IHC versus Taqman® CNV was assessed.

**Results:**

Concordance between pathologists (PTEN loss vs no loss) on IHC assessment was 37/51 (73%). In specimens with concordant IHC assessment, concordance between IHC and Taqman® copy number in PTEN loss assessment was 25/37 (68%).

**Conclusion:**

Assessment PTEN loss in colorectal cancer is limited by the inter-observer variability of IHC, and discordance of CNV with loss of protein expression. An understanding of the genetic mechanisms of PTEN loss and implementation of improved and standardized methodologies of PTEN assessment are required to clarify the role of PTEN as a biomarker in colorectal cancer.

## Background

Survival for patients with metastatic colorectal cancer (mCRC) has improved significantly over the past 15 years, largely due to improved systemic treatment options [1]. The availability of biological agents inhibiting angiogenesis via vascular endothelial growth factor (VEGF) pathway and targeting oncogenic cell signaling via epidermal growth factor receptor (EGFR) have contributed to these improved outcomes. With the advent of new treatment options has come the search for predictive biomarkers to assist selection of patients most likely to benefit from these agents and equally to avoid toxicity and expense for those who are unlikely to benefit. RAS gene mutation (KRAS and NRAS) remains the only validated predictive marker in mCRC and predicts for lack of benefit to anti-EGFR monocloncal antibodies (MoAbs) cetuximab and panitumumab [2-6]. In addition to RAS, mutation of genes involved in downstream EGFR signaling pathways Ras/Raf/MAPK and PI3K/AKT have been proposed to confer resistance to anti-EGFR MoAbs [6-8]. Specifically, mutations in BRAF and PIK3CA genes are likely to predict resistance to anti-EGFR MoAbs although analyses on retrospective cohorts have been conflicting [7,9-11].

PTEN is an important negative regulator of PI3K/AKT pathway and controls cell proliferation, survival and angiogenesis. Loss of PTEN function leads to persistent activation of the PI3K pathway and has been observed in breast, prostate, glioblastoma, endometrial and colon cancers [12,13]. Loss of PTEN function, generally evaluated by loss of PTEN protein expression, has been suggested as both prognostic in mCRC [8,14,15] and a predictive biomarker for response to anti-EGFR MoAbs [16,17] although results remain conflicting and difficult to interpret.

Several crucial factors make testing and interpretation of PTEN difficult. Loss of PTEN function results from several genetic mechanisms including small scale PTEN gene mutations (point mutations, insertions, small deletions), allelic loss at chromosome 10 and epigenetic silencing via hypermethylation of the PTEN promoter region [18]. PTEN gene mutations are relatively uncommon, occurring in 2.2-12% [6,19,20] of CRC specimens and therefore account for only a small proportion of loss of PTEN expression on IHC staining (19-54%) [8,17,19,21]. This highlights the role of alternate mechanisms such as allelic loss and epigenetic silencing in impairing protein expression. These mechanisms are likely to coexist leading to a “second hit” and resulting in bi-allelic inactivation [13,16].

Further complicating the situation, the frequency of loss of PTEN expression increases from progression from normal colonic mucosa to adenoma, primary CRC and ultimately metastasis [21]. The resultant discordance between primary and metastatic CRC has been consistently demonstrated [16,17,22,23]. This highlights a major limitation of cohort studies assessing the predictive value of PTEN loss in mCRC patients that have used only primary CRC specimens for analysis [24-27].

Clearly the role of PTEN is more complex than KRAS gene mutation where a single identifiable mechanism (activating mutation), largely concordant between primary and secondary tumours, confers near complete resistance to anti-EGFR MoAbs. Understanding this complexity is central to interpreting the current literature relating to PTEN and its potential role as a predictive biomarker. Recently reported cohorts of mCRC patients receiving anti-EGFR MoAbs have used PTEN loss of IHC expression to report loss of PTEN function. While this represents the functional outcome of several genetic mechanisms of PTEN loss, IHC relies on subjective interpretation and has the potential for inter reporter variation. Furthermore there is variability over the definition of 'loss of PTEN’ based on IHC scoring. In the largest cohort of mCRC patients, PTEN loss was defined as no staining in any cells at any intensity [8], while other groups have used various cut-offs of reduced PTEN expression [17,28-30]. Others groups investigating the predictive role of PTEN have assessed PTEN allelic loss by fluorescent in situ hydridization (FISH) [31], PTEN mutation [6,19,20], and PTEN promoter methylation [16] but concordance with loss of PTEN expression by IHC remains unclear. The consistent demonstration of PTEN as a useful biomarker in mCRC has been, and will continue to be, limited until assessment of PTEN loss is better clarified and validated.

Our group undertook an analysis of PTEN status in the AGITG MAX study of mCRC patients to identify the rate of inter-observer variability in IHC assessment and the rate of discordance between IHC and PCR assessment of PTEN status.

## Methods

### Patients and study design

The MAX study design and eligibility criteria have been reported previously [32].

**Figure 1:**
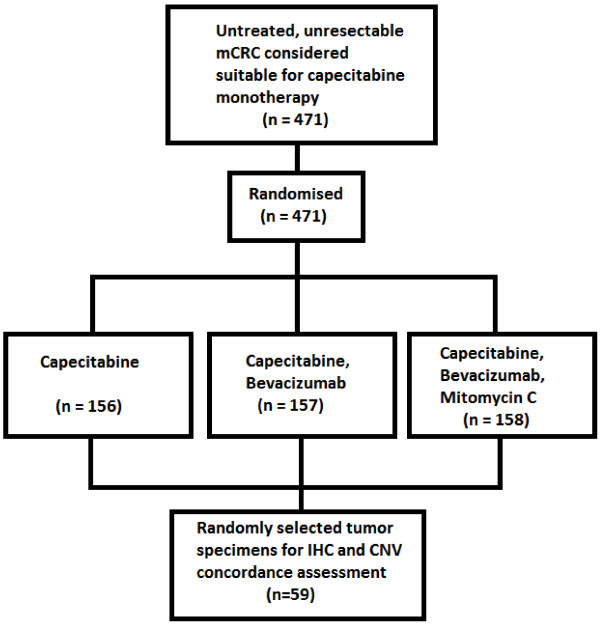
CNV = copy number variation, IHC = immunohistochemistry, mCRC = metastatic colorectal cancer.

The primary objective of this Phase III randomized trial was to evaluate the effect of adding bevacizumab to capecitabine (with or without mitomycin C) on progression free survival (PFS) among patients receiving first line chemotherapy for mCRC. Four hundred and seventy-one patients were enrolled between July 2005 and June 2007. We have used the TaqMan® Copy Number Assay (Life Technologies, Carlsbad, CA) to assess for PTEN allelic loss and have previously reported that loss of PTEN copy number was not prognostic nor predictive of outcome in the MAX trial cohort [33]. In this report we randomly selected 59 tumor samples to explore the potential inter-observer variability between pathologists assessment of PTEN loss of expression by IHC, and concordance of IHC PTEN loss and the Taqman® results (see Figure 1). Ethics approval for translational studies was obtained centrally.

### Tumour collection and processing

Formalin-fixed, paraffin-embedded (FFPE) samples of tumor tissue from archival specimens collected at the time of diagnosis were retrieved from storage at hospital pathology departments. For Copy Number PCR, genomic DNA was extracted from FFPE tissue sections with the use of the QIAamp DNA FFPE tissue kit (Qiagen, Valencia, CA). Manual micro-dissection was performed on samples with less than 80% malignant cells when visualized by microscopy. The same tissue blocks were used to make tissue microarrays (TMAs) and were assessed for PTEN expression by IHC. Researchers who assessed PTEN IHC expression were blinded to the PCR results.

### Immunohistochemistry

**Figure 2:**
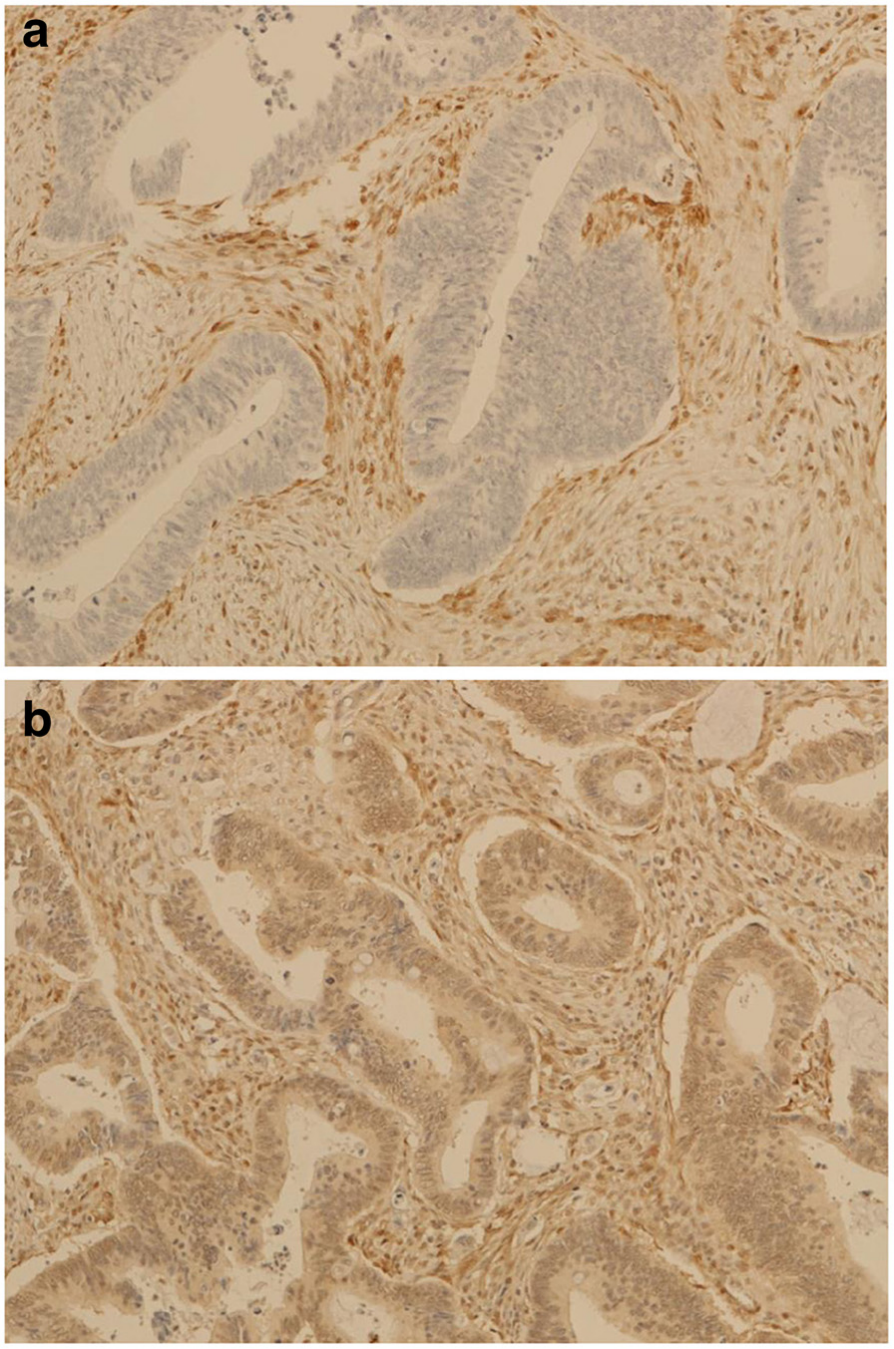
Examples of immunohistochemical assessment of PTEN (a) IHC negative (b) IHC positive.

Immunohistochemical staining was carried out on TMAs using the PTEN monoclonal antibody 6H2.1 (Dako, Glostrup, Denmark) that has been used previously [34-36]. Essentially, tissue sections (3 μm) as TMAs were deparaffinised by heating the slides at 55-60°C for 2 hours, then soaking in xylene and hydrating by passing through a graded series of ethanol to water. Antigen retrieval was carried out by microwaving the slides in target retrieval solution pH 9 (DAKO). Endogenous peroxidase was quenched by incubating the slides in Peroxidazed I reagent (Biocare Medical, Concord, CA) for 5 min and background staining was blocked by incubation in Background Sniper reagent (Biocare Medical). Slides were stained using a 1:100 dilution of PTEN primary antibody 6H2.1 and detected using the MACH 3™ mouse HRP polymer detection system according to the manufacturer’s protocol (Biocare Medical). Slides were counterstained in methyl green (Sigma). The TMAs contained 3 sections taken from the same core. Each section was assessed by 2 blinded pathologists (JC and AR) and a majority score was determined for each pathologist (3 IHC readings). PTEN staining was mostly cytoplasmic. Intensity was scored on a four-tier system: 0, no staining; 1, weak; 2, moderate; and 3, strong. Loss of PTEN was defined as majority score 0 (Figure 2). The pathologist’s majority scores were compared directly for an IHC concordance rate. Specimens concordant on IHC were used for IHC versus TaqMan concordance rate.

### PTEN copy number variation

The PTEN TaqMan® copy number assay (Hs03007912_cn, Life Technologies) was performed using 10 ng DNA in quadruplicate PCR. The primers provided in the assay were entirely within exon 9 of *PTEN*, at cytoband 10q23.31a, location Chr.10:89727445 on NCBI build 37 (Life Technologies). The assay is a duplex PCR for the PTEN gene and the reference gene, RNaseP (normaliser), set up according to the supplier’s protocol and run on the Rotorgene 6000 real time PCR instrument (Qiagen, Valencia, CA). The results are calculated as a ratio relative to a 2-copy control using the 2^-∆∆Ct^ method (Rotorgene software), and multiplied by 2 to give the copy number. We tested DNA from colon cancer cell lines to determine the reproducibility of the assay and to select cell lines to use as copy number controls. HT29 (ATCC) is known to have 3 copies of chromosome 10 as determined by spectral karyotyping and comparative genomic hybridization [37] and was used as the primary control sample for 3 PTEN copies. Cell lines LIM2405, LIM1899 (both a kind gift from The Ludwig Institute, Melbourne) and HT29 were tested in quadruplicate and repeated in 3 separate PCR assays. The assay was both precise and reproducible - the means for LIM2405, LIM1899 and HT29 were 1.08 SEM 0.04, 2.07 SEM 0.03 and 2.96 SEM 0.07 respectively, and the coefficient of variation (CV) from run to run was 2.4%, and intra-assay CV was between 0.12% and 0.99%. These cell lines were therefore used as 1-, 2- and 3-copy controls respectively. Our group has previously described quantification of PTEN gene copy number on cell lines LIM2405 and LIM1899 [33]. For the patients’ DNA, loss of PTEN was defined as ≤1.5 copies, no loss was >1.5 copies.

## Results

Fifty-nine tumor specimens were analysed for loss of copy number by Taqman® and for loss of protein expression by IHC. Eight samples were found to contain no tumor tissue and were excluded from further analysis.

### Immunohistochemistry

Two blinded pathologists assessed 51 specimens independently for PTEN protein expression with IHC. Pathologist JC assessed 29/51 (57%) as having PTEN expression loss, while pathologist AR assessed 17/51 (33%) as having loss of PTEN expression. Concordance between pathologists on final IHC assessment (PTEN loss/no loss) was 37/51 (73%), indicating in 14/51 (27%) of specimens there was discordance in the final assessment of IHC PTEN loss (Table 1)*.*

**Table 1:** Loss of PTEN by IHC and CNV

**PTEN loss**	**PTEN copy number**		**IHC score**	**Pathologist**	
				JC	AR
			NT	5	0
Loss	≤1.5	26	0	29	17
No loss	>1.5	30	+1, +2, +3	22	39
Total		56		56	56

*CNV* copy number variation, *IHC* immunohistochemistry, *NT* no tumour identified, *PTEN* phosphatase and tensin homologue.

### Taqman® copy number PCR

Using a PTEN Taqman® copy number assay, 25/51 specimens (49%) had ≤1.5 copy number and were thus classified as PTEN loss.

### Concordance between IHC and Taqman®PCR

The 37 specimens with concordant IHC assessment were included in the IHC versus Taqman*®* PCR concordance analysis. Fifteen specimens had PTEN loss on IHC of which 10 (67%) also had PTEN allelic loss on Taqman® PCR. Seventeen specimens had PTEN allelic loss on Taqman® PCR of which 10 (58%) had PTEN loss on IHC. Fifteen specimens had preserved PTEN on both IHC and Taqman® PCR analysis. Overall concordance between IHC and Taqman® copy number in PTEN loss assessment was 25/37 (68%) (Table 2).

**Table 2:** Concordance of PTEN loss between IHC and Taqman copy number

**Taqman/IHC**	**PTEN loss (0 staining score)**	**NO PTEN loss (+1, +2, +3)**	
PTEN loss (≤1.5)	10	7	17
No PTEN loss (>1.5)	5	15	20
Total	15	22	37

*IHC* Immunohistochemistry, *PTEN* phosphatase and tensin homologue.

Shaded squares = discordant results.

## Discussion

In this validation study of PTEN assessment in CRC we evaluated inter-observer variability in PTEN assessment with IHC and subsequently the discordance of PTEN assessment between IHC and PCR based methodologies. IHC assessment yielded rates of PTEN loss of 33% and 57% between two pathologists, while Taqman® PCR demonstrated 49% of specimens contained PTEN allelic loss. Our analysis provides particular insight into the relationship between PTEN protein expression and allelic loss. Specifically how is protein expression maintained in the setting of allelic loss, and why do samples show absence of PTEN expression despite allelic loss?

In samples with PTEN allelic loss 41% maintained protein expression. Of these specimens all had IHC staining intensity of 1+ suggesting possibly a reduced level of PTEN protein. The maintenance of protein expression in these cases is likely due to the remaining functional PTEN allele, which allows transcription of a normal PTEN protein. In cases of PTEN haploinsufficiency (monoallelic loss) whether protein expression is reduced and whether such reduction confers a growth advantage is unknown. Sood et al. also demonstrated monoallelic PTEN dysfunction (by mutation or promoter methylation) resulted in loss of protein expression in only 38% of samples, while biallelic inactivation resulted in loss of PTEN expression in 80% of cases [16]. Ali et al. reported a higher PTEN expression loss of 71% in samples with a single PTEN gene mutation, though allelic loss and methylation were not assessed [19].

In our cohort 25% of cases without PTEN allelic loss demonstrated complete absence of PTEN expression on IHC. These findings confirm alternative genetic mechanisms, beyond allelic loss, are responsible for loss of PTEN protein expression. Several authors have undertaken more comprehensive analysis of PTEN status on CRC specimens and provide an important insight into the often coexisting genetic mechanisms of PTEN dysfunction. Goal et al. demonstrated hypermethylation of the PTEN promoter region occurred in 10/132 (7.6%) sporadic CRC specimens, with a higher rate (19.1%) in microsatellite unstable CRCs. PTEN mutations coexisted in 4/10 (40%) of hypermethylated PTEN specimens. Eighty percent of patients with promoter hypermethylation had reduced (+1) or loss of PTEN protein expression and in the 3 cases of complete loss of PTEN staining, promoter hypermethylation coexisted with PTEN mutation or allelic loss [13]. Nassif et al. assessed allelic loss and PTEN mutation in 41 primary CRC specimens, finding 15 (37%) contained one or both aberrations. Nine of these cases contained biallelic inactivation [12]. Perrone et al. assessed both allelic loss by FISH and PTEN mutation in 32 mCRC samples. Thirteen percent had reduced PTEN copy number, 10% contained PTEN mutations and only one specimen (3%) had coexisting copy number loss and PTEN mutation [38]. These results suggest a comprehensive analysis of all known mechanisms of PTEN dysfunction, including determination of biallelic inactivation is likely to provide the most robust determination of PTEN dysfunction.

Alternatively, focusing on loss of protein expression at least represents the functional outcome of any such genetic insult. We have demonstrated the current limitations of IHC for this purpose. In our cohort, IHC assessment of PTEN loss by two pathologists was 33% and 57%, with overall concordance of 73%. As this was designed as a validation subset we did not ask the two pathologists to discuss the results that were not concordant, nor seek a further opinion, methods commonly described in papers reporting PTEN IHC aimed at reducing the apparent discordance rate [10]. In all 14 cases of IHC discordance one pathologist assessed the tumor as having no PTEN staining (score 0) with the other pathologist recording weak (score 1) staining. This highlights the subjective nature of IHC scoring, and the inherent difficulty in arbitrary scoring of a continuous (staining intensity) trait. The problematic inter-observer variability of PTEN IHC is frequently reported in the literature [18,39] but rarely quantified. Recently Sangale et al. evaluated PTEN IHC using 5 potential PTEN antibodies on standardized cell lines. With the selected optimal antibody a validation study of 50 human tumor specimens produced 100% concordance between three independent pathologists using dichotomous reporting of PTEN loss [30]. The significant inter-observer variability in PTEN IHC has also been demonstrated in prostate and breast cancer also allowing for optimized assays [40,41].

Overall the current literature highlight the difficulty in accurately measuring PTEN function to date; measurement of a single genetic insult, while minimizing inter-observer variability, does not capture the often coexisting mechanisms required for biallelic inactivation. The use of IHC, while potentially a better measure of PTEN function, is observer dependent and there remains a lack of consensus on optimal methodology and scoring.

Given the limitations of PTEN assessment discussed here, it is not surprising reports of the predictive value of PTEN as a biomarker in CRC remain conflicting [8,17,27,31]. In contrast, there appears more consistency in the prognostic role of PTEN in colorectal cancer. Loss of PTEN expression in primary CRC has been associated with poor prognostic pathological features [19] as well as higher rates of metachronous liver metastases [42]. Several retrospective cohort studies have demonstrated reduced survival in patients with loss of PTEN by IHC [8,14,15,43]. This is however, in contrast to our cohort of patients from the MAX clinical trial where loss of PTEN copy number by Taqman® PCR was not prognostic [33].

## Conclusion

The lack of standardization in assessing loss of PTEN function appears to have contributed significantly to the conflicting results from retrospective cohort studies. Further elucidation of PTEN as a potential biomarker for colorectal cancer relies on defining PTEN loss of function and standardizing analytical methods and scoring systems. Future studies assessing PTEN function may be better served by obtaining a more comprehensive analysis of PTEN function by assessing PTEN mutation, hypermethylation of PTEN promoter, PTEN allelic loss and protein expression on each specimen. An alternative approach may be to explore improved methods of measuring reduced protein expression beyond IHC, given reduced or absent protein expression should reflect the functional outcome of PTEN loss irrespective of the genetic mechanism. Immuno-PCR may provide an option of combining the protein-specific capability of antibodies with the objective quantification of real-time PCR [44]. This will be the focus of a future study.

## Abbreviations

CNV: Copy number variation; CV: Coefficient of variability; EGFR: Epidermal growth factor receptor; FFPE: Formalin-fixed, paraffin-embedded; FISH: Fluorescent in situ hybridization; IHC: Immunohistochemistry; mCRC: Metastatic colorectal cancer; MoAbs: Monocloncal antibodies; PTEN: Phosphatase and tensin homologue; TMAs: Tissue microarrays; VEGF: Vascular endothelial growth factor.

## Competing interests

The authors declare that they have no competing interest.

## Author contributions

CH drafted the manuscript. JH conceived the study and coordinated all laboratory aspect of the study. VB participated in the design of the study and assisted with writing of manuscript. JW was involved in performing the DNA isolation and copy number PCR. NT contributed to trial conception and design as well as assisting with manuscript preparation. JC undertook the immunohistochemistry of the specimens and contributed to study design. AR undertook the immunohistochemistry of the specimens and contributed to study design. CL undertook data analysis and statistical analysis. TP conceived the study and participated in its design and coordination of all aspects of the study including drafting of the manuscript. ART designed study and assisted with manuscript. All authors read and approved the final manuscript.

## Pre-publication history

The pre-publication history for this paper can be accessed here:

http://www.biomedcentral.com/1471-2407/14/128/prepub
